# Phenolic Profile, Antioxidant Activity and Amino Acid Composition of Moringa Leaves Fermented with Edible Fungal Strains

**DOI:** 10.3390/foods11233762

**Published:** 2022-11-22

**Authors:** Anna Starzyńska-Janiszewska, Bożena Stodolak, Carmen Fernández-Fernández, Barbara Mickowska, Vito Verardo, Ana María Gómez-Caravaca

**Affiliations:** 1 Department of Biotechnology and General Technology of Food, Faculty of Food Technology, University of Agriculture in Krakow, Balicka 122, 30-149 Kraków, Poland; 2 Department of Analytical Chemistry, Faculty of Sciences, University of Granada, Av. de Fuentenueva s/n, 18071 Granada, Spain; 3 Malopolska Centre of Food Monitoring, Faculty of Food Technology, University of Agriculture in Krakow, 30-149 Kraków, Poland; 4 Department of Nutrition and Food Science, Campus of Cartuja, University of Granada, 18071 Granada, Spain; 5 Institute of Nutrition and Food Technology ‘José Mataix’, Biomedical Research Centre, University of Granada, Avda del Conocimiento s/n, 18100 Armilla, Spain

**Keywords:** *Moringa oleifera* Lam., phenolic compounds, amino acids, antioxidant activity, solid-state fermentation

## Abstract

Solid-state fermentation (SSF) is widely recognised as a technique to increase the bioactive potential and nutritional value of plant materials. However, the effect of this biotreatment differs for individual substrates. This study aimed to evaluate the impact of SSF with filamentous fungi (*Rhizopus*, *Aspergillus*, and *Neurospora*) on a moringa leaf phenolic profile, antioxidant activity, and amino acid composition. A total of 43 phenolic compounds were determined in the dried leaves analysed by HPLC-ESI-TOF-MS. The leaves contained 11.79 mg/g of free phenolics: flavonols (80.6%, mainly quercetin and kaempferol glycosides), hydroxycinnamic acid derivatives (12.3%), vitexin and vicenin (6.9%), and a small amount of lignan (isolariciresinol isomers). The result of the 1-day fermentation was a slight enhancement in the concentration of individual free phenolics (flavones) and the antioxidant activity of the leaves. However, extending the incubation period caused a significant decrease in those parameters and cannot be recommended for obtaining a food fortificant from moringa leaves. In contrast, the 3-day fermentation with *N. intermedia* led to a 26% average accumulation of individual amino acids. Therefore, the SSF with *Neurospora* can be a promising method for improving the nutritional composition of moringa leaves and needs further investigation.

## 1. Introduction

Moringa (*Moringa oleifera* Lam.) is a tropical plant grown in Africa, Asia, and Europe. It is widely cultivated for the nutritious properties of various parts—seeds, fruits, flowers, and leaves, which can be incorporated into food and feeds. Moringa leaves are exceptionally rich in protein compared to other edible leaves, but the exact concentration of this compound is variable, between 15% and 30%, and depends on genetic and environmental factors. Other compounds present in moringa leaves include polyunsaturated fatty acids, fibre, minerals, and bioactive compounds (vitamins and phenolics) [1]. Moringa leaf flour has been used as a food fortificant, for example, in gluten-free bread, cheese, and sour cream [2,3], and as a vegetable snack ingredient [4].

Solid-state fermentation (SSF) is widely recognised as an effective technique for enhancing the bioactive potential and nutritional value of edible plant materials. The enzymatic activity of microorganisms enriches the product in valuable microbial metabolites and partially hydrolyses substrate macronutrients, thus increasing the availability of bioactive ingredients [5]. Few studies on the submerged and SSF of moringa leaves have been conducted recently. The published literature concern the commonly used microorganisms, such as *Rhizopus oligosporus*, *Aspergillus niger*, *Bacillus subtilis*, *Candida utilis*, and *Lactobacillus* sp. [6,7,8,9,10]. Shi et al. (2021) demonstrated the effect of mixed-strain SSF with fungi (*C. utilis*, *A. niger*) and bacteria (*B. subtilis*) on reducing the concentration of anti-nutritional compounds (tannins, phytate, and glucosinolates), and improving the nutritional parameters (crude protein, total amino acids) in moringa leaves [8]. In another study, a 3-day mixed-culture SSF with the microorganisms mentioned above was reported as optimal for producing various metabolites (e.g., oligosaccharides, amino acids, organic acids, and myoinositol) [9]. Moreover, lactic acid fermentation was proven to increase the bioavailability of calcium in moringa leaves [10]. Data concerning the influence of SSF on the antioxidant activity, total phenolics, and total flavonoids in moringa leaves indicate higher effectiveness of the shorter fermentation periods, one to three days [7,9,11], but varying results are also presented [6]. However, no previous studies have analysed the changes in the profile of free phenolics occurring during the fermentation process. This information seems important because flavonoids and phenolic acid derivatives are the most abundant bioactive compounds in moringa leaves [12].

When investigating the effectiveness of biotreatments for processing plant materials, SSF with the edible fungal strains traditionally used for food production is particularly interesting. Our earlier studies concerned the application of koji (*Aspergillus oryzae*), tempeh (*Rhizopus* sp.), and oncom (*Neurospora* sp.) moulds for increasing the nutritional and bioactive value of substrates, such as legumes, pseudo-cereals, and edible oil cakes [13,14,15]. These moulds are generally considered a safe species, which do not synthesise toxic secondary metabolites and have a long history of safe use in food production [16,17,18].

The aim of the present study was to assess the effectiveness of SSF with *Rhizopus oligosporus*, *Rhizopus oryzae*, *Aspergillus oryzae*, and *Neurospora intermedia* by increasing the bioactive potential of moringa leaves, with a special focus on the free phenolic profile analysed using HPLC-ESI-TOF-MS. Moreover, the impact of the fermentation on the amino acid composition was assessed because the protein quality is a very important feature of moringa leaves. The fermented moringa leaves could be used as a bioactive food supplement with enhanced soluble phenolic concentrations and as a nutritious product with an increased amino acid score.

## 2. Materials and Methods

### 2.1. Materials

Moringa (*Moringa oleifera*) dried leaves were purchased from Apycsa (Cajíz, province of Malaga, Spain, 36°45′35″ N 4°11′06″ W). The leaves were ground into uniform powder in an ultra-centrifugal mill, ZM 200 (Retsch GmbH, Haan, Germany), reaching an average particle size of 0.5 mm, and then stored at −20 °C until its use.

### 2.2. Microbial Strains

*Rhizopus oligosporus* ATTC 64063, *R. oryzae* CBS 372.63, *Aspergillus oryzae* CBS 673.92 and *Neurospora intermedia* CBS 131.92 were grown on a potato extract agar for 12 days at 24 °C. The spore suspension of each strain in sterile saline (8 g/L, with 0.01 g/L peptone and 0.1 mL/L Tween 80) was filtered through nylon net filters (ø 11 μm, Millipore, Cork, Ireland) to remove mycelium. The spore density was quantified by spore-counting in a Thoma chamber.

### 2.3. Autoclaving and Fermentation

Portions of 25 g dried moringa leaves were placed in Erlenmeyer flasks (three replicates for each fermentation period) and autoclaved (121 °C, 20 min). After cooling, the samples were hydrated to a 60% moisture content with sterile distilled water and inoculated with 1 × 10^9^ spore suspensions of the individual strains. The flasks were incubated at 30 °C. The fermentation was performed for 1, 3, 8 and 16 days. The fungal growth was stopped by steaming the products (10 min). The autoclaved and fermented samples were lyophilized and stored at 20 °C until they were analysed.

The analyses were performed on the dried, autoclaved, and fermented moringa leaves.

### 2.4. Analyses

#### 2.4.1. Antioxidant Methods

The antioxidant parameters were measured in extracts obtained with a 70:30 (*v*/*v*) ethanol/water solution.

The method of Swain and Hillis [19] was used to determine the Folin–Ciocalteu Reacting Substances (FCRS) (mg gallic acid/g dry matter (DM)).

The 2,2′-azino-bis(3-ethylbenzothiazoline-6-sulfonic acid (ABTS) radical scavenging activity (SA-ABTS˙^+^) was determined according to the Cano, Acosta, and Arnao methods [20]. Briefly, before the analysis, the stock ABTS˙^+^ solution (20 mg ABTS in 2.6 mL 0.0049 mol/L K_2_S_2_O_8_ and 2.6 mL distilled water) was diluted with phosphate buffer to obtain an absorbance value of 0.7 at 734 nm. A sample extract (200 μL) was added to 2 mL of the ABTS˙^+^ solution, mixed, and incubated at room temperature in the dark. After 7 min, the absorbance was measured at 734 nm, with a 70:30 (*v*/*v*) ethanol/water solution.

The scavenging activity of ABTS˙^+^ was expressed as μmol Trolox equivalents/g DM.

The hydroxyl radical neutralisation activity (SA-˙OH) was measured as described by Marambe et al. [21]. Briefly, 50–200 μL extract was made up to 1125 μL with potassium phosphate buffer (20 mmol/L, pH 7.4). Then, the following components were added: 40 μL 0.5 mmol/L FeCl_3_, 42 μL 2.4 mmol/L EDTA, 140 μL, 0.02 mol/L deoxyribose, 10 μL 0.01 mol/L ascorbic acid, and 142 μL 1 mmol/L H_2_O_2_. After incubation at 37 °C for 1 h, 1 mL of 1% (*w*/*v*) thiobarbituric acid solution (TBA) and 1 mL of 2.8% (*w*/*v*) trichloroacetic acid solution (TCA) were added, and the samples were placed in a boiling water bath for 20 min. After centrifugation, the absorbance was measured at 532 nm against a blank (all the reagents, with the TBA and TCA added prior to incubation at 37 °C). The SA-˙OH was expressed as the half maximal inhibitory concentration (IC_50_), which refers to the mg DM of the sample used for the extraction that is required for the 50% inhibition of the free radicals under reaction conditions. The lower the IC_50_ value, the higher the hydroxyl radical scavenging activity.

#### 2.4.2. Determination of Phenolic Compounds

The extraction of the phenolic compounds was performed according to Rodríguez-Pérez et al. [12]. Briefly, 0.5 g of a sample was extracted with 25 mL of ethanol/water solution (1:1 *v*/*v*) using an ultrasonic bath (Bandelin, Sonorex, RK52, Berlin, Germany), which worked at a frequency of 35 kHz. The supernatants collected after centrifugation were dried in a rotary evaporator under vacuum at 40 °C. The dried residue was redissolved in 2 mL of methanol/water 1:1 (*v*/*v*), filtered through a 0.22 μm syringe nylon filter, and kept at − 18 °C until the analysis. Each extraction was performed in triplicate.

The determination of the phenolic compounds was conducted according to the gradient and the column proposed by Rodriguez-Pérez et al. [12]. An ACQUITY Ultra Performance LC system (Waters Corporation, Milford, MA, USA), coupled to an electrospray ionisation (ESI) source operating in the negative mode and a time-of-flight (TOF) mass detector (Waters Corporation, Milford, MA, USA), was used for this purpose.

#### 2.4.3. Amino Acid Profile

The amino acid profile was determined according to the method described by Moore and Stein [22,23,24]. The samples were hydrolysed in liquid 6 mol/L HCl containing 0.5% phenol at 110 °C for 24 h under an argon atmosphere. After lyophilization, the obtained hydrolysates were dissolved in sodium citrate buffer (pH 2.2) and filtered through a 0.45 μm syringe filter. An amino acid analyser with a strong cation ion exchanger was used. Amino acids were eluted with a sodium-citrate buffers gradient and spectrophotometrically detected at 570 and 440 nm after post-column derivatization with ninhydrin, according to the standard manufacturer’s protocol (Amino acid analyser AAA400. User manual. Ingos s.r.o. Praha 2007). Sulphur-containing amino acids were analysed as oxidation products (after performing acid oxidation followed by the standard hydrolysis procedure with HCl); therefore, asparagine and glutamine were determined as aspartic and glutamic acids. Tryptophan was not determined, as it is destroyed during acid hydrolysis. For calibration of the amino acid analyser, the amino acid standard solution was used (Sigma, St. Louis, MO, USA). Data processing was performed using the software of the chromatographic device (Chromulan, Pikron, Czech Republic).

#### 2.4.4. SDS-PAGE Electrophoresis

For direct protein extraction, the samples were mixed with the denaturing and reducing sample buffer (125 mmol/L Tris-HCl pH 6.8, 4% SDS, 20% *v*/*v* glycerol, DTT 50 mg/mL), boiled at 100 °C for 5 min, and centrifuged. Then, SDS-PAGE electrophoresis was performed using the method of Schägger–von Jagow [25]. The PageRuler Prestained Protein Ladder and Spectra Multicolor Low Range Protein Ladder (Thermo Fisher Scientific, Waltham, MA, USA) were used as molecular weight protein markers. Gels were run in the Mini PROTEAN Tetra Cell electrophoresis equipment (Bio-Rad Laboratories, Hercules, CA, USA) and stained with Coomassie Brilliant Blue R-250.

#### 2.4.5. Glucosamine Determination

A 0.5 g sample or 10 mg standard *N*-acetyl-D-glucosamine was hydrolysed in 10 mol/L HCl for 16 h at 20 °C and then autoclaved in 2 mol/L HCl for 2 h at 130 °C. Next, the released glucosamine (g/100 g DM) was measured using the colorimetric method with 3-methyl-2-benzothiazolone hydrazone hydrochloride [26]. The background amount of glucosamine in the substrate was subtracted from the total glucosamine content in the fermented products to calculate the fungal glucosamine concentration.

#### 2.4.6. Dry Matter Determination

DM was estimated using a moisture analyser (WPS 110S, Radwag, Radom, Poland).

### 2.5. Statistical Analysis

The results reported in the present study were statistically analysed using a one-way or two-way analysis of variance (ANOVA) with the Fisher post hoc test (Statistica for Windows ver. 13.1 software, Statsoft, Tulsa, OK, USA). The differences were considered significant at a *p* ≤ 0.05. The results were expressed as a mean ± standard error of the mean (SEM).

## 3. Results and Discussion

### 3.1. The Characteristics of the Fungal Growth during the Fermentation of Moringa Leaves

**Figure 1:**
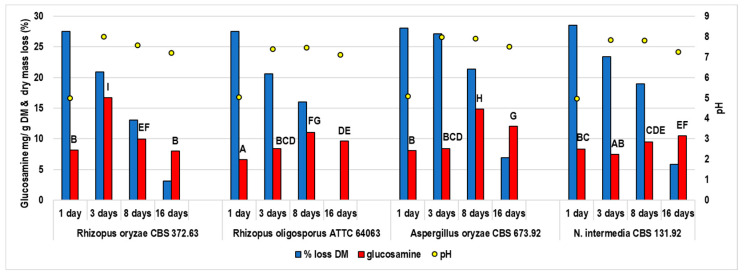
The concentration of glucosamine, dry matter loss, and pH during the fermentation of moringa leaves.

**Figure 2:**
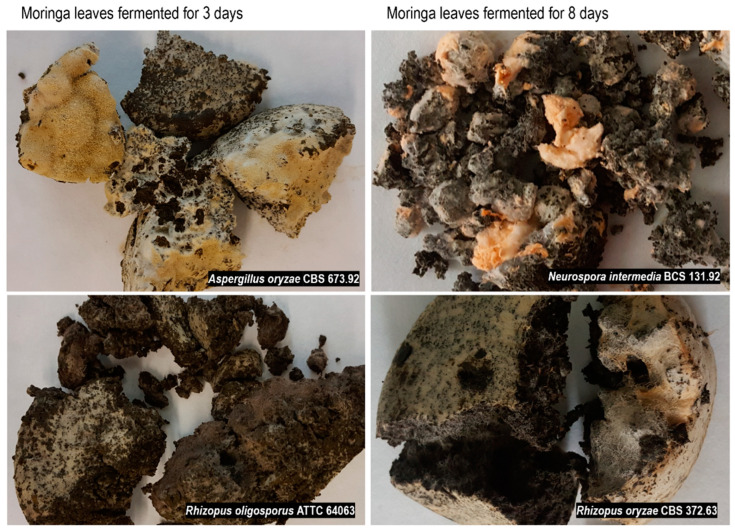
Fermented moringa leaves.

The fermentation was performed for 16 days; samples were collected after 1, 3, 8, and 16 days. The changes in the substrate pH, DM loss, and fungal glucosamine content were monitored during incubation (Figure 1). Moringa leaves were an appropriate substrate for the fungi, as samples fermented for 1 day contained 6.2–8.29 mg glucosamine per gram of DM. Mycelium was also visible on the substrate surface, except for the *R. oligosporus* strain. The products obtained after 3 days were compact and overgrown with mycelium, with visible spores on the surface. Figure 2 shows the representative samples obtained in the experiment. The pH markedly increased after 3 days of fermentation, from around 4 (pH of the inoculated substrate) to 7–8, then remained within this range. The DM loss suggests that the metabolic activity of the moulds decreased after 8 and 16 days of incubation.

### 3.2. Antioxidant Activity in the Dried, Autoclaved, and Fermented Moringa Leaves

The concentration of the FCRS measured in extracts from the dried moringa leaves was 38.91 mg/g (Table 1), which falls within the range previously reported [1]. The results of the ABTS˙^+^ scavenging method (SA-ABTS˙^+^) (505 µmol trolox/g) and ˙OH neutralisation assay (the half maximal inhibitory concentration, IC_50_ 0.8 mg) demonstrate that the antiradical activity of the substrate was high. Organic extracts from moringa leaves are recognised as a rich source of antioxidants, which among them are quercetin, kaempferol, rutin, and other phenolic compounds [1,27]. In the present study, the extracts were obtained using 70% ethanol because this solvent was previously proven appropriate for obtaining a high-quality antioxidant extract from raw moringa leaves [27].

Autoclaving resulted in a slight decrease in the FCRS (by 6%) and SA-ABTS˙^+^ (by 12%). It also resulted in a doubling of the sample dose necessary for 50% ˙OH neutralisation.

The observed changes in SA-ABTS˙^+^, due to the fermentation, were highly correlated with the FCRS (linear correlation coefficient = 0.930, *p* < 0.05). Feitosa et al. (2020) showed that in moringa leaves fermented with *A. niger*, such a correlation is not always the case and depends on the composition of individual extracts [7]. The expected effect of the fermentation on the antioxidant potential of the substrate is the improvement in extractability of such compounds from the plant matrix as a result of the action of the microbial hydrolytic enzymes. The *Rhizopus* and *Aspergillus* strains are shown to produce enzymes, such as pectinase, amylase, cellulase, β-glucosidase and feruloyl esterase [28]. The samples fermented for 1 day were characterised by a higher concentration of the FCRS and SA-ABTS˙^+^, up to a maximum of 20–25% (*A. oryzae* and *R. oligosporus*). However, the prolongation of incubation gradually diminished the antioxidant activity of the products (Table 1 and Table S1). The antioxidant potential of the samples fermented for 16 days was lowered by approximately 40% compared to the autoclaved leaves. A similar tendency—an improvement in the total phenols after 1 day of incubation, followed by a decrease—was previously observed in the 7-day fermentation of autoclaved moringa leaves with the *A. niger* strain [7]. The SSF of moringa leaves with *Bacillus subtilis* was also shown to be effective only for up to 2 days, after which the total phenols and ABTS˙^+^ scavenging activity decreased [11]. A different dynamic was reported in a study where *R. oligosporus* enriched the moringa leaves with total phenols for up to 6 days of a total of 9-days incubation, but, in this case, the culture medium also contained molasses [6]. The effect of the biotreatment on the hydroxyl radical neutralisation was positive, as the fermentation generally enhanced the activity of the autoclaved samples (Table 1 and Table S1). The products obtained after the 1 and 3-day incubations were characterised by IC_50_ lower by approximately 27% and 36%, respectively, than the autoclaved sample. However, the longer fermentation stages did not improve this parameter further.

Regarding the microorganisms studied, the products obtained with the *R. oryzae* strain contained more FCRS and scavenged ABTS˙^+^ more effectively than the others (Table 1 and Table S1). The lowest antioxidant activity was measured for the moringa leaves fermented with N. intermedia. In a previous study, a decrease in the antiradical activity of methanol extracts from rice bran was noted after SSF with *R. oryzae* [29]. In contrast, the incubation of moringa leaves with *R. oligosporus* and *A. niger* was beneficial for the antioxidant potential of the samples [6,7]. The A. niger strain enriched the moringa leaves in the FCRS by as much as 112% and 136%, depending on the initial substrate moisture concentration (50% and 70%, respectively) [7].

The effect of the fermentation on the FCRS and antiradical potential of dried moringa leaves observed in this study was clearly strain- and time-dependent (Table S1). However, the plant matrix used as the fermentation substrate is probably the most important factor. As proven in a study focused on the fungus–substrate interaction during the SSF of agro-residues (rice straw, banana, orange and pomegranate peels, empty pea pods) to produce antioxidants, during the selection of components, the choice of mould species is less important than the choice of substrate [30].

### 3.3. Determination of Phenolic Compounds in Dried, Autoclaved, and Fermented Samples by Using HPLC-ESI-TOF-MS

A total of 43 phenolic compounds were determined in the moringa leaf samples analysed using HPLC-ESI-TOF-MS. They were identified according to the literature [12] and according to the molecular formula and the mass-to-charge ratio (*m*/*z*). Among them, 15 compounds belonged to hydroxycinnamic acid and derivatives, 7 to flavones and derivatives, 19 to flavonols and derivatives, and 2 to lignans (Table 2, Figure S1). Most of the identified compounds were consistent with the data reported by Rodríguez-Pérez et al. [12] with the use of the HPLC–ESI–QTOF–MS technique.

The dried moringa leaves had 11.79 mg/g of free phenolics, the majority of which were flavonols and derivatives (FVL) (80.6%) (Table 2). Within this fraction, the predominant groups were 3-O-glycosides of quercetin (66.8%) and kaempferol (23.6%). This result confirms the earlier studies considering flavonoids and, among them, quercetin and its derivatives as dominant free phenolics in moringa leaves [1]. The leaves also contained isorhamnetin glucosides (0.246 mg/g). The next largest fraction, which accounted for 12.3% of the free phenolics in the dried moringa leaves, was the hydroxycinnamic acid derivatives (HCA). The most abundant HCA was caffeoylquinic acid (88.65%). The other free phenolics in the dried leaves were flavones and derivatives (FL): vitexin and vicenin (6.9% of the free phenolics) and a small amount of lignan—isolariciresinol isomers in a glycoside form.

Autoclaving caused, on average, a 19% increase in the concentration of free phenolics because a high-temperature treatment allows for the breakdown of the linkage between bound phenolic forms and the moringa leaf cell walls. The phenolic profile changed due to a higher percentage of flavones (180% increase, 16% of the total free phenolic), hydroxycinnamic acids (63% increase, 17% of the total), and lignans (240% increase, 0.6% of the total). In contrast, autoclaving had different results on the individual compounds within the FVL fraction; however, their total did not change after the treatment.

The profile of the free phenolics was measured in the extracts from the samples fermented for 1 and 3 days, as they were characterised by a higher antioxidant potential than the later fermentation stages (Table 1 and Table S1). The products fermented for 1 day were characterised by a similar (*Rhizopus* strains) or slightly lower sum of the total free phenolics than the autoclaved leaves. A different tendency was found only within the FV fraction, where an increase in the vicenin and/or vitexin concentrations was noted in the case of *R. oryzae* (by 23%), *R. oligosporus* (by 15%), and *A. oryzae* (by 8%). These findings can be considered advantageous because apigenin and its glucosides were recognised as important components of moringa leaves due to their beneficial health-promoting activity and low intrinsic toxicity [12]. Previously, increased concentrations of vitexin and vicenin were found in millet fermented with *Lactobacillus* sp. and yeasts [31]. Higher contents of these compounds in moringa leaves could result from the use of other components of the substrate by the moulds during fermentation. However, it is worth mentioning that some fungal strains (*Fusarium solani* G6, *Dichotomopilus funicola* Y3) were reported to produce vitexin [32,33].

After 3 days of fermentation, a drastic decrease in the content of free phenolics was found in the products. In the autoclaved sample, the total free phenolics content decreased by 43% (*R. oligosporus*), 70% (*R. oryzae*), 74% (*N. intermedia*), and even 97% (*A. oryzae*). Compared to the 1-day fermentation, the profile of the free phenolics in the samples fermented for 3 days was characterised by a higher percentage of HCA, up to approximately 20% in the case of the *Rhizopus* strains, and FL, up to 55% in the case of *A. oryzae* and *N. intermedia*. The FVLs constitute the fraction decomposed during fermentation to a higher extent, and their percentage in the total phenolics dropped to 55.5% in the moringa leaves fermented with *R. oryzae* and to 23–37% in the case of the other strains. A significant decrease in the soluble flavonoids of teff grain fermented with mushroom fungi *Ganoderma elucidum* and *Pleurotus ostreatus* has been reported [34]. When considering the quercetin: kaempferol ratio, a change was noted in the samples fermented with *A. oryzae*, in which the quercetin derivatives accounted for 80% of the FVLs (Table 2). The moringa leaves fermented with this fungus were also characterised by the highest percentage of lignan fraction in the profile, 7%.

Changes in the free phenolics during SSF result from the mobilisation of phenolics from their complexes within the plant matrix and their further rearrangements by microorganisms. Our results clearly show that the free phenolic compounds in moringa leaves were mostly utilised by the moulds in the course of fermentation. Fungal strains can biotransform flavonoids, e.g., the oxidation and cleavage of the quercetin C-ring by quercetinase or flavonol 2,4-dioxygenase, as reported for the *Aspergillus* strains [28]. The decrease in free phenolic content during the later fermentation stages may also be due to their oxidation and polymerization by the lignifying and tannin-forming peroxidases (laccase, manganese peroxidase, lignin peroxidase) [35].

### 3.4. Amino Acid Profile and Protein in the Dried, Autoclaved, and Fermented Moringa Leaves

The dried moringa leaves had 25% protein, based on the total amino acid content (Table 3), which is within the typical range [1] and confirms that this material is a valuable protein source. All the essential amino acids were at a higher concentration than in the recommended scoring pattern [36]. The moringa leaf protein was especially rich in aromatic amino acids and threonine (Table 4).

Autoclaving induced a small change in the amino acid content and profile. The exception was lysine, which decreased in autoclaved leaves by 15%, and methionine, which increased by 51%.

The amino acid profile was determined in the samples with fully developed fungal cultures—after 3 and 8 days of fermentation (Table 3, Figure S2). The fermentation resulted in minor changes in the protein concentration in the leaves, with two exceptions. The leaves fermented with *R. oryzae* contained less protein, 16% on average. In contrast, fermentation with *N. intermedia* resulted in a significant protein enrichment in the leaves, approximately 21%. Consequently, the concentrations of the individual amino acids in these products also increased. The amino acid profile of protein in moringa fermented with *N. intermedia* and the autoclaved samples was similar, with 46% essential amino acids (Table 4). These results indicate that *N. intermedia* activity can be more effective than a mixed-culture fermentation of moringa leaves with *Bacillus subtilis* and *Aspergillus niger*, where a 17% increase in protein concentration was found [8]. Fermentation with the *Neurospora* strains previously proved beneficial for the concentration and composition of protein in quinoa seeds and flaxseed oil cake [13,15], as well as lignocellulosic substrates [37]. As shown by Shojaosadati et al. [37], the protein enrichment of sugar beet pulp, wheat bran, and citrus waste was correlated with cellulose consumption by the *Neurospora* strain, which was capable of effective cellulase synthesis.

**Figure 3:**
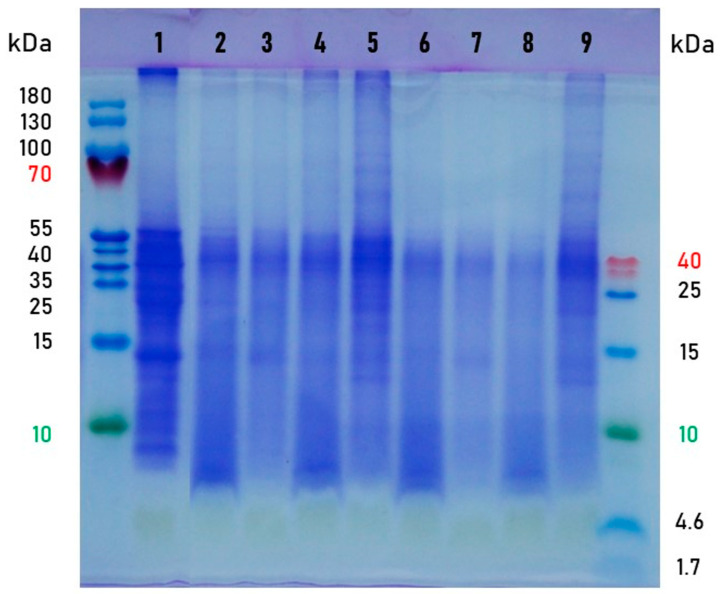
SDS-PAGE analysis of protein profiles in moringa leaves. Sample description: the dried leaves (1); the fermented leaves: *Rhizopus oryzae*—3 days (2) and 8 days (6), *Rhizopus oligosporus*—3 days (3) and 8 days (7), *Aspergillus oryzae*—3 days (4) and 8 days (8), *Neurospora intermedia*—3 days (5) and 8 days (9).

The dried leaves and the fermented products (3 and 8 days) were subjected to SDS-PAGE to analyse the influence of SSF on the moringa leaf protein (Figure 3). The molecular weight of the main protein fractions in the dried moringa leaves was approximately between 55 kDa and 25 kDa. The protein profiles of the fermented samples were characterised by more distinct bands of smaller molecular masses (peptides below 10 kDa) and a gradual disappearance of protein bands of higher molecular weights, clearly visible in the dried leaves. The decrease in protein sizes and peptide appearance can be considered advantageous for the digestibility of moringa protein. The leaves fermented with the *N. intermedia* were richer in protein and peptides than the other bioprocessed samples, which is consistent with the results of the total amino acid content (Table 3). The appearance of more distinct bands of protein, larger than 55 kDa, reveals the metabolic activity of the mould synthesising its own proteins. The tendency observed in the present study is consistent with that described by Shi et al. [8] for a 5-day fermentation of moringa leaves with a mixed-culture inoculum consisting of *Aspergillus niger*, *Candida utilis*, and *Bacillus subtilis*.

## 4. Conclusions

The present study demonstrates the effectiveness of SSF with edible mould strains as a method for increasing the bioactive potential and nutritional value of moringa leaves. The changes in the profile of the free phenolic compounds were evaluated throughout the fermentation process using HPLC-ESI-TOF-MS. The concentrations of the free phenolic fractions in the moringa leaves were significantly increased after the autoclaving treatment, which preceded the fermentation stage. The leaves that were fermented for 1 day with *Rhizopus oryzae*, *R. oligosporus*, and *Aspergillus oryzae* had a higher concentration of vicenin, vitexin, or both. However, despite the increased antioxidant potential, no improvement in the total content of free phenolics was found after fermentation. Prolongation of the fermentation caused a drastic decrease in the content of free phenolics, accompanied by a diminished antiradical activity, and cannot be recommended for enhancing the bioactive potential of moringa leaves. In contrast, fermentation with *Neurospora intermedia* significantly enriched the leaves in proteins of an advantageous amino acid score. Therefore, fermentation with *N. intermedia* could be a promising tool for upgrading the nutritional value of moringa leaves. However, further studies concerning the effect of this biotreatment on the digestibility of proteins and other parameters, such as vitamins and dietary fibre, are required.

## Figures and Tables

**Table 1:** The antioxidant potential of moringa leaves—dried, autoclaved, and fermented with the studied microorganisms.

			FCRS (mg/g DM)	SA-ABTS˙^+^ (µmol Trolox/g DM)	SA-˙OH (IC_50_)
Dried moringa			38.91 ± 0.82 ij	505.44 ± 9.53 l	0.80 ± 0.07 a
Autoclaved moringa			36.44 ± 0.59 h	442.32 ± 6.82 j	1.60 ± 0.06 i
Fermented moringa	Strain	Time (days)			
	*Rhizopus oryzae* CBS 372.63	1	42.91 ± 1.01 l	462.06 ± 6.93 k	0.97 ± 0.05 bcd
		3	29.70 ± 0.64 g	316.57 ± 5.23 g	0.84 ± 0.02 ab
		8	26.69 ± 0.73 f	306.72 ± 15.70 fg	0.87 ± 0.06 abc
		16	25.71 ± 0.45 e	297.71 ± 9.30 f	0.93 ± 0.12 abdc
	*Rhizopus oligosporus* ATCC 64063	1	39.23 ± 0.60 jk	533.67 ± 14.99 m	1.21 ± 0.04 fgh
		3	30.44 ± 0.63 g	343.45 ± 2.93 h	0.98 ± 0.01 bcd
		8	20.33 ± 0.54 b	243.52 ± 4.07 b	1.02 ± 0.05 cde
		16	18.46 ± 0.26 a	216.70 ± 3.27 a	1.16 ± 0.24 efg
	*Aspergillus oryzae* CBS 673.92	1	38.12 ± 1.13 i	552.29 ± 11.30 n	1.16 ± 0.10 efg
		3	21.68 ± 0.44 c	257.17 ± 5.95 cd	1.30 ± 0.11 gh
		8	22.92 ± 0.42 d	271.72 ± 6.60 e	1.20 ± 0.05 fgh
		16	21.85 ± 0.54 b	267.17 ± 7.63 de	1.16 ± 0.07 efg
	*Neurospora intermedia* CBS 131.92	1	40.15 ± 0.46 k	396.28 ± 8.83 i	1.34 ± 0.07 h
		3	23.24 ± 0.45 d	269.56 ± 7.01 e	0.97 ± 0.05 bcd
		8	22.39 ± 0.29 cd	251.64 ± 9.69 bc	1.04 ± 0.13 de
		16	18.95 ± 0.93 a	226.36 ± 6.03 a	1.08 ± 0.16 def

One-factor analysis of variance and Fisher post hoc test were applied. Data are shown as the mean ± SE. Mean values within a column followed by different letters differ significantly (*p* ≤ 0.05). FCRS—Folin–Ciocalteu reacting substances; SA-ABTS˙^+^—ABTS˙^+^-scavenging activity; SA-˙OH—˙OH-scavenging activity; DM: dry matter; IC_50_: half maximal inhibitory concentration.

**Table 2:** The quantification of phenolic compounds (mg/g DM) in moringa leaves—dried, autoclaved, and fermented—with the studied microorganisms at each time of fermentation (1 and 3 days).

		Dried Moringa	Autoclaved Moringa	*Rhizopus oryzae* CBS 372.63		*Rhizopus oligosporus* ATCC 64063		*Aspergillus oryzae* CBS 673.92		*Neurospora intermedia* CBS 131.92	
P^a^	Compound	-		1 Day	3 Days	1 Day	3 Days	1 Day	3 Days	1 Day	3 Days
	Hydroxycinnamic acids and derivatives										
1	Glucopyranosyl-caffeoylquinic acid isomer a	0.028 ± 0.001 a	0.118 ± 0.008 c	0.107 ± 0.000 b	<LOD	0.106 b ± 0.000 b	<LOD	0.107 ± 0.003 b	<LOD	0.107 ± 0.005 b	<LOD
4	Glucopyranosyl-caffeoylquinic acid isomer b	<LOD	0.069 ± 0.010 b	0.049 ± 0.000 a	<LOD	0.048 ± 0.001 a	<LOD	0.051 ± 0.001 a	<LOD	0.051 ± 0.001 a	<LOD
6	Glucopyranosyl-caffeoylquinic acid isomer c	0.009 ± 0.001 a	0.091 ± 0.003 d	0.082 ± 0.000 c	<LOD	0.078 ± 0.002 bc	<LOD	0.075 ± 0.002 b	<LOD	0.077 ± 0.006 bc	<LOD
9	1-Caffeoylquinic acid	<LOD	0.226 ± 0.011 e	0.085 ± 0.001 c	0.082 ± 0.000 c	0.070 ± 0.003 b	0.169 ± 0.001 d	0.072 ± 0.001 b	0.018 ± 0.001 a	0.085 ± 0.002 c	<LOQ
2	3-Caffeoylquinic acid	1.180 ± 0.021 e	0.355 ± 0.033 d	0.204 ± 0.006 c	0.064 ± 0.002 a	0.155 ± 0.000 b	0.158 ± 0.003 b	0.149 ± 0.003 b	<LOD	0.193 ± 0.009 c	<LOD
7	4-Caffeoylquinic acid	0.080 ± 0.007 b	0.472 ± 0.034 g	0.250 ± 0.001 f	0.081 ± 0.003 b	0.175 ± 0.005 cd	0.206 ± 0.001 e	0.164 ± 0.004 c	0.017 ± 0.001 a	0.202 ± 0.007 de	<LOQ
3	3-p-Coumaroylquinic acid isomer a	0.074 ± 0.006 b	0.119 ± 0.004 d	0.140 ± 0.005 f	0.076 ± 0.001 b	0.129 ± 0.003 e	0.090 ± 0.000 c	0.114 ± 0.004 d	<LOD	0.116 ± 0.001 d	0.058 ± 0.002 a
5	3-p-Coumaroylquinic acid isomer b	0.059 ± 0.003 b	0.166 ± 0.000 f	0.186 ± 0.003 g	0.103 ± 0.001 c	0.168 ± 0.002 f	0.123 ± 0.003 d	0.161 ± 0.007 ef	<LOD	0.152 ± 0.007 e	0.035 ± 0.001 a
10	4-p-Coumaroylquinic acid isomer a	0.003 ± 0.000 a	0.167 ± 0.011 f	0.132 ± 0.003 e	0.091 ± 0.003 c	0.111 ± 0.001 d	0.102 ± 0.002 cd	0.101 ± 0.006 cd	<LOD	0.108 ± 0.004 d	0.058 ± 0.003 b
12	4-p-Coumaroylquinic acid isomer b	0.004 ± 0.001 a	0.252 ± 0.012 g	0.216 ± 0.000 f	0.133 ± 0.005 c	0.193 ± 0.008 e	0.151 ± 0.008 d	0.190 ± 0.008 e	<LOD	0.183 ± 0.002 e	0.040 ± 0.002 b
13	5-p-Coumaroylquinic acid isomer a	<LOD	0.071 ± 0.007 b	0.050 ± 0.001 a	0.113 ± 0.004 d	0.048 ± 0.000 a	0.104 ± 0.000 c	0.052 ± 0.001 a	<LOD	0.051 ± 0.002 a	0.068 ± 0.004 b
14	5-p-Coumaroylquinic acid isomer b	<LOD	0.074 ± 0.001 b	0.051 ± 0.000 a	0.118 ± 0.000 d	0.049 ± 0.001 a	0.107 ± 0.004 c	0.052 ± 0.002 a	<LOD	0.052 ± 0.001 a	<LOQ
8	3-Feruloylquinic acid	0.016 ± 0.001 a	0.069 ± 0.001 f	0.069 ± 0.001 f	0.042 ± 0.000 b	0.065 ± 0.002 e	0.052 ± 0.000 c	0.061 ± 0.003 d	<LOD	0.061 ± 0.001 d	<LOD
15	4-Feruloylquinic acid	<LOD	0.068 ± 0.007 d	0.058 ± 0.002 c	0.042 ± 0.001 a	0.056 ± 0.001 c	0.048 ± 0.000 ab	0.057 ± 0.002 a	<LOD	0.054 ± 0.001 bc	<LOD
17	5-Feruloylquinic acid	<LOD	0.047 ± 0.004 b	0.036 ± 0.000 a	0.049 ± 0.001 b	0.035 ± 0.001 a	0.051 ± 0.001 b	0.037 ± 0.001 a	<LOD	0.037 ± 0.001 a	<LOD
	Flavones and derivatives										
11	Vicenin-2 (apigenin 6,8-C-dihexoside) isomer a	<LOQ	0.408 ± 0.032 b	0.748 ± 0.013 f	0.474 ± 0.023 c	0.707 ± 0.016 ef	0.603 ± 0.025 d	0.674 ± 0.030 e	0.047 ± 0.001 a	0.600 ± 0.026 d	0.715 ± 0.001 ef
16	Vicenin-2 (apigenin 6,8-C-dihexoside) isomer c	<LOQ	1.085 ± 0.033 d	1.217 ± 0.006 f	0.893 ± 0.014 b	1.146 ± 0.041 e	1.011 ± 0.028 b	1.046 ± 0.029 cd	0.179 ± 0.002 a	1.000 ± 0.025 c	1.083 ± 0.025 d
18	Vicenin-2 (apigenin 6,8-C-dihexoside) isomer d	<LOQ	0.056 ± 0.006 e	0.056 ± 0.002 e	0.041 ± 0.001 b	0.052 ± 0.003 de	0.051 ± 0.001 de	0.049 ± 0.004 cd	0.007 ± 0.001 a	0.043 ± 0.003 bc	0.050 ± 0.001 de
19	Vicenin-2 (apigenin 6,8-C-dihexoside) isomer e	0.025 ± 0.001 f	0.020 ± 0.001 e	<LOQ	0.019 ± 0.001 de	0.009 ± 0.001 b	0.017 ± 0.002 cd	<LOQ	<LOQ	0.014 ± 0.001 c	0.001 ± 0.000 a
26	Vicenin-2 (apigenin 6,8-C-dihexoside) isomer f	<LOQ	0.087 ± 0.006 e	0.075 ± 0.005 d	0.007 ± 0.001 b	0.074 ± 0.003 d	0.045 ± 0.000 c	0.075 ± 0.002 d	<LOQ	0.072 ± 0.002 d	0.006 ± 0.001 a
21	(Vitexin) Apigenin 8-C-glucoside isomer a	0.541 ± 0.002 e	0.472 ± 0.003 d	0.521 ± 0.031 e	0.177 ± 0.002 b	0.485 ± 0.010 d	0.355 ± 0.013 c	0.461 ± 0.015 d	<LOQ	0.456 ± 0.017 d	0.124 ± 0.003 a
22	(Vitexin) Apigenin 8-C-glucoside isomer b	0.270 ± 0.016 f	0.142 ± 0.012 de	0.154 ± 0.000 e	0.044 ± 0.002 a	0.138 ± 0.004 de	0.086 ± 0.006 b	0.126 ± 0.001 cd	<LOQ	0.121 ± 0.004 c	0.048 ± 0.001 a
	Flavonols and derivatives										
24	Quercetin rutinoside (rutin)	0.673 ± 0.023 g	0.424 ± 0.007 e	0.467 ± 0.006 f	0.041 ± 0.001 a	0.390 ± 0.014 d	0.154 ± 0.004 b	0.350 ± 0.006 c	<LOQ	0.363 ± 0.005 c	0.033 ± 0.004 a
25	Quercetin 3-O-β-D-glucoside/Isoquercetin	1.347 ± 0.086 d	2.491 ± 0.003 g	2.144 ± 0.105 f	0.844 ± 0.016 c	2.010 ± 0.047 e	1.272 ± 0.018 d	1.975 ± 0.069 e	0.071 ± 0.017 a	2.087 ± 0.027 ef	0.660 ± 0.041 b
27	Quercetin hydroxy-methylglutaroyl galactoside isomer a	<LOQ	0.126 ± 0.012 c	0.123 ± 0.004 c	<LOQ	0.113 ± 0.003 bc	0.031 ± 0.001 a	0.105 ± 0.004 b	<LOD	0.103 ± 0.004 b	<LOQ
29	Quercetin 3-O-malonylglucoside	0.353 ± 0.002 f	0.109 ± 0.008 b	0.192 ± 0.005 e	<LOQ	0.177 ± 0.001 d	0.044 ± 0.000 a	0.131 ± 0.005 c	<LOD	0.139 ± 0.006 c	<LOQ
30	Quercetin 3-O-acetyl glucoside isomer a	3.973±0.322 e	2.189 ± 0.180 c	3.021 ± 0.110 d	0.029 ± 0.001 a	2.821 ± 0.013 d	1.054 ± 0.023 b	2.297 ± 0.080 c	<LOQ	2.420 ± 0.071 c	0.090 ± 0.011 a
32	Quercetin 3-O-acetyl glucoside isomer b	0.619 ± 0.038 c	0.933 ± 0.105 d	0.688 ± 0.042 c	<LOQ	0.630 ± 0.030 c	0.288 ± 0.003 b	0.627 ± 0.013 c	<LOQ	0.632 ± 0.013 c	0.013 ± 0.001 a
33	Quercetin 3-O-acetyl glucoside isomer c	0.047 ± 0.003 b	0.109 ± 0.015 e	0.100 ± 0.005 de	<LOQ	0.088 ± 0.001 d	0.007 ± 0.001 a	0.068 ± 0.003 c	<LOD	0.064 ± 0.003 c	<LOQ
34	Quercetin 3-O-acetyl glucoside isomer d	<LOD	0.042 ± 0.002 b	0.095 ± 0.005 f	<LOQ	0.084 ± 0.004 e	0.016 ± 0.001 a	0.072 ± 0.004 d	<LOD	0.055 ± 0.003 c	<LOQ
42	Quercetin	<LOQ	0.130 ± 0.012 c	0.136 ± 0.004 c	0.048 ± 0.000 b	0.132 ± 0.002 c	0.170 ± 0.002 d	0.129 ± 0.004 c	0.008 ± 0.001 a	0.132 ± 0.005 c	<LOD
40	Quercetin derivative	<LOQ	0.069 ± 0.008 d	0.048 ± 0.003 b	<LOD	0.047 ± 0.002 b	0.020 ± 0.001 a	0.047 ± 0.002 b	<LOD	0.058 ± 0.000 c	<LOQ
28	Kaempferol 3-O-glucoside/Kaempferol 3-O-hexose/Astragalin	<LOQ	0.569 ± 0.031 h	0.333 ± 0.021 b	0.527 ± 0.012 g	0.380 ± 0.016 c	0.521 ± 0.002 f	0.454 ± 0.011 de	0.010 ± 0.001 e	0.421 ± 0.001 d	0.482 ± 0.023 e
35	Kaempferol 3-O-acetyl glucoside isomer a	2.051 ± 0.167 e	1.198 ± 0.024 c	1.719 ± 0.096 d	0.020 ± 0.000 a	1.617 ± 0.076 d	0.605 ± 0.011 b	1.305 ± 0.033 c	<LOQ	1.328 ± 0.024 c	0.078 ± 0.006 a
36	Kaempferol 3-O-acetyl glucoside isomer b	<LOQ	0.002 ± 0.000 a	0.011 ± 0.001 c	<LOD	0.008 ± 0.001 b	<LOQ	0.003 ± 0.001 a	<LOD	0.004 ± 0.001 a	<LOQ
38	Kaempferol 3-O-acetyl glucoside isomer c	0.187 ± 0.002 b	0.635 ± 0.029 e	0.545 ± 0.001 d	<LOQ	0.529 ± 0.031 cd	0.197 ± 0.002 b	0.532 ± 0.008 cd	<LOQ	0.504 ± 0.006 c	0.012 ± 0.001 a
39	Kaempferol 3-O-acetyl glucoside isomer d	<LOQ	0.069 ± 0.004 e	0.054 ± 0.001 d	<LOQ	0.046 ± 0.004 c	<LOQ	0.036 ± 0.001 b	<LOD	0.022 ± 0.002 a	<LOQ
43	Kaempferol	<LOQ	0.037 ± 0.001 d	0.031 ± 0.001 c	0.030 ± 0.000 c	0.030 ± 0.002 c	0.025 ± 0.000 ab	0.026 ± 0.000 b	<LOQ	0.023 ± 0.000 a	<LOQ
31	Isorhamnetin 3-O-glucoside	0.009 ± 0.000 a	0.096 ± 0.008 c	0.043 ± 0.004 a	<LOQ	0.042 ± 0.003 a	0.017 ± 0.001 b	0.041 ± 0.000 a	<LOQ	0.042 ± 0.002 a	<LOQ
37	Isorhamnetin 3-acetyl-glucoside isomer a	0.237 ± 0.010 c	0.044 ± 0.005 a	0.081 ± 0.003 b	<LOQ	0.072 ± 0.003 b	<LOQ	0.047 ± 0.003 a	<LOD	0.049 ± 0.001 a	<LOQ
41	Isorhamnetin 3-acetyl-glucoside isomer b	<LOQ	0.018 ± 0.001 c	0.008 ± 0.000 b	<LOD	0.007 ± 0.001 b	<LOQ	0.008 ± 0.000 b	<LOD	0.003 ± 0.000 a	<LOQ
	Lignans										
20	Isolariciresinol glycoside (lariciresinol glycoside) isomer a	0.010 ± 0.001 a	0.050 ± 0.001 f	0.046 ± 0.001 e	0.033 ± 0.000 c	0.046 ± 0.001 e	<LOD	0.044 ± 0.001 e	0.028 ± 0.000 b	0.044 ± 0.001 e	0.039 ± 0.002 d
23	Isolariciresinol glycoside (lariciresinol glycoside) isomer b	0.016 ± 0.001 a	0.037 ± 0.001 f	0.037 ± 0.001 ef	0.032 ± 0.000 c	0.036 ± 0.000 e	0.031 ± 0.000 c	0.030 ± 0.000 b	<LOD	0.034 ± 0.000 d	<LOD

P^a^—Peak numbers assigned according to the overall elution order; LOD—limit of detection; LOQ—limit of quantification; One-factor analysis of variance and Fisher post hoc test were applied. Data are shown as the mean ± SE. Mean values within a row followed by different letters differ significantly (*p* ≤ 0.05).

**Table 3:** The quantification of amino acids (g/100 g DM) in moringa leaves—dried, autoclaved, and fermented—with the studied microorganisms at each fermentation time point (3 and 8 days).

Compound	Dried Leaves	Autoclaved Leaves	*Rhizopus oryzae * CBS 372.63		*Rhizopus oligosporus* ATCC 64063		*Aspergillus oryzae* CBS 673.92		*Neurospora intermedia* CBS 131.92	
	-		3 Days	8 Days	3 Days	8 Days	3 Days	8 Days	3 Days	8 Days
Essential amino acids										
Isoleucine	1.22 ± 0.02 b	1.27 ± 0.01 bc	1.04 ± 0.04 a	1.06 ± 0.02 a	1.30 ± 0.11 c	1.22 ± 0.01 bc	1.21 ± 0.02 b	1.23 ± 0.01 bc	1.52 ± 0.08 d	1.54 ± 0.07 d
Leucine	2.31 ± 0.03 ab	2.42 ± 0.03 bc	1.83 ± 0.08 a	1.82 ± 0.03 a	2.49 ± 0.22 bc	2.35 ± 0.01 c	2.19 ± 0.05 b	2.19 ± 0.04 b	2.89 ± 0.15 d	2.83 ± 0.14 d
Lysine	1.56 ± 0.02 f	1.32 ± 0.02 cd	1.10 ± 0.04 a	1.05 ± 0.02 a	1.37 ± 0.12 de	1.23 ± 0.01 b	1.27 ± 0.02 bc	1.25 ± 0.04 bc	1.63 ± 0.08 f	1.43 ± 0.06 e
Methionine	0.41 ± 0.06 a	0.62 ± 0.02 ef	0.53 ± 0.01 bcd	0.48 ± 0.02 ab	0.62 ± 0.01 ef	0.60 ± 0.01 de	0.57 ± 0.07 cde	0.48 ± 0.13 abc	0.75 ± 0.01 g	0.68 ± 0.08 fg
Phenylalanine	1.60 ± 0.02 c	1.64 ± 0.02 c	1.29 ± 0.05 a	1.26 ± 0.02 a	1.65 ± 0.15 c	1.56 ± 0.02 bc	1.49 ± 0.04 b	1.47 ± 0.03 b	1.95 ± 0.11 d	1.91 ± 0.09 d
Threonine	1.26 ± 0.01 cd	1.30 ± 0.02 d	1.00 ± 0.04 a	0.99 ± 0.02 a	1.27 ± 0.11 cd	1.20 ± 0.01 bc	1.20 ± 0.02 bc	1.17 ± 0.01 b	1.58 ± 0.08 e	1.58 ± 0.08 e
Valine	1.51 ± 0.02 bc	1.59 ± 0.02 c	1.23 ± 0.05 a	1.26 ± 0.02 a	1.54 ± 0.14 bc	1.46 ± 0.01 b	1.48 ± 0.03 b	1.50 ± 0.01 bc	1.85 ± 0.10 d	1.87 ± 0.08 d
Histidine	0.64 ± 0.01 d	0.65 ± 0.01 d	0.61 ± 0.03 abc	0.57 ± 0.01 a	0.70 ± 0.06 e	0.65 ± 0.01 d	0.62 ± 0.01 bcd	0.58 ± 0.01 ab	0.85 ± 0.04 g	0.78 ± 0.04 f
Nonessential amino acids										
Alanine	1.79 ± 0.01 d	1.86 ± 0.02 d	1.53 ± 0.07 b	1.64 ± 0.03 c	1.64 ± 0.14 bc	1.54 ± 0.01 ab	1.59 ± 0.03 abc	1.60 ± 0.01 abc	1.97 ± 0.10 e	2.01 ± 0.10 e
Arginine	1.66 ± 0.01 d	1.65 ± 0.02 cd	1.34 ± 0.05 ab	1.24 ± 0.02 a	1.66 ± 0.15 d	1.56 ± 0.01 c	1.42 ± 0.02 b	1.40 ± 0.01 b	2.25 ± 0.11 f	1.89 ± 0.09 e
Aspartic acid	2.57 ± 0.03 bc	2.67 ± 0.03 bc	2.36 ± 0.10 a	2.22 ± 0.04 a	2.71 ± 0.23 c	2.53 ± 0.02 b	2.53 ± 0.04 b	2.53 ± 0.02 b	3.17 ± 0.16 d	3.08 ± 0.16 d
Cysteine	0.25 ± 0.04 a	0.31 ± 0.01 b	0.39 ± 0.00 d	0.37 ± 0.01 cd	0.34 ± 0.00 bc	0.34 ± 0.00 bc	0.34 ± 0.04 bc	0.30 ± 0.07 ab	0.46 ± 0.00 e	0.45 ± 0.05 e
Glutamic acid	3.64 ± 0.03 a	3.72 ± 0.04 a	3.64 ± 0.15 a	3.57 ± 0.06 a	3.96 ± 0.34 b	3.73 ± 0.03 ab	3.77 ± 0.06 ab	3.76 ± 0.04 ab	4.75 ± 0.25 c	4.83 ± 0.24 c
Glycine	1.38 ± 0.02 bc	1.46 ± 0.01 c	1.26 ± 0.06 a	1.22 ± 0.02 a	1.55 ± 0.13 d	1.46 ± 0.01 c	1.35 ± 0.02 b	1.31 ± 0.01 ab	1.81 ± 0.09 e	1.79 ± 0.09 e
Serine	1.33 ± 0.01 cd	1.37 ± 0.01 d	1.06 ± 0.04 a	1.04 ± 0.02 a	1.25 ± 0.11 bc	1.19 ± 0.01 b	1.22 ± 0.02 b	1.19 ± 0.01 b	1.55 ± 0.08 e	1.56 ± 0.08 e
Tyrosine	1.02 ± 0.01 b	1.01 ± 0.01 b	0.90 ± 0.03 a	0.87 ± 0.01 a	1.12 ± 0.10 c	1.04 ± 0.01 bc	1.10 ± 0.06 c	1.05 ± 0.06 bc	1.45 ± 0.07 d	1.40 ± 0.07 d
Proline	1.32 ± 0.01 cd	1.37 ± 0.02 d	1.07 ± 0.05 a	1.00 ± 0.02 a	1.29 ± 0.12 cd	1.20 ± 0.01 b	1.28 ± 0.02 bc	1.29 ± 0.02 cd	1.57 ± 0.09 e	1.54 ± 0.08 e
Total g/100 g	25.47 ± 0.23 bcd	26.23 ± 0.26 cd	22.17 ± 0.88 a	21.67 ± 0.38 a	26.45 ± 2.22 d	24.85 ± 0.18 bc	24.63 ± 0.49 b	24.29 ± 0.17 b	32.02 ± 1.58 e	31.20 ± 1.59 e

One-factor analysis of variance and Fisher post hoc test were applied. Data are shown as the mean ± SEM. Mean values within a row followed by different letters differ significantly (*p* ≤ 0.05).

**Table 4:** The quality of protein (g amino acid/100 g protein) of moringa leaves—dried, autoclaved, and fermented—with the studied microorganisms at each fermentation time point (3 and 8 days).

Compound	Dried Leaves	Autoclaved Leaves	*Rhizopus oryzae* CBS 372.63		*Rhizopus oligosporus* ATCC 64063		*Aspergillus oryzae* CBS 673.92		*Neurospora intermedia* CBS 131.92		RSP1	RSP2
	-		3 Days	8 Days	3 Days	8 Days	3 Days	8 Days	3 Days	8 Days		
Histidine	2.52 ± 0.03 abc	2.47 ± 0.04 a	2.73 ± 0.12 d	2.65 ± 0.06 bcd	2.64 ± 0.21 bcd	2.62 ± 0.02 bcd	2.50 ± 0.05 ab	2.38 ± 0.04 a	2.67 ± 0.13 cd	2.51 ± 0.12 ab	2.0	1.6
Isoleucine	4.78 ± 0.08 a	4.83 ± 0.05 ab	4.67 ± 0.19 a	4.87 ± 0.08 ab	4.90 ± 0.43 ab	4.91 ± 0.03 ab	4.93 ± 40.10 ab	5.07 ± 0.05 b	4.75 ± 0.24 a	4.91 ± 0.22 ab	3.2	3.0
Leucine	9.08 ± 0.10 cd	9.22 ± 0.10 cd	8.24 ± 0.34 a	8.41 ± 0.14 ab	9.42 ± 0.84 cd	9.44 ± 0.06 d	8.91 ± 0.21 bc	8.99 ± 0.15 cd	9.02 ± 0.47 cd	9.08 ± 0.43 cd	6.6	6.1
Lysine	6.14 ± 0.07 d	5.05 ± 0.06 bc	4.97 ± 0.19 bc	4.85 ± 0.09 ab	5.16 ± 0.44 c	4.96 ± 0.03 bc	5.16 ± 0.09 c	5.13 ± 0.17 bc	5.09 ± 0.25 bc	4,56 ± 0.21 a	5.7	4.8
Methionine + Cysteine	2.59 ± 0.38 a	3.56 ± 0.12 bc	4.15 ± 0.05 d	3.91 ± 0.13 cd	3.61 ± 0.05 bc	3.78 ± 0.04 cd	3.69 ± 0.44 bcd	3.22 ± 0.84 b	3.78 ± 0.03 cd	3.65 ± 0.41 bcd	2.7	2.3
Phenylalanine + Tyrosine	10.27 ± 0.07 abc	10.11 ± 0.12 abc	9.88 ± 0.39 ab	9.85 ± 0.13 a	10.46 ± 0.94 abc	10.47 ± 0.10 abc	10.51 ± 0.39 bc	10.38 ± 0.37 abc	10.65 ± 0.56 c	10.63 ± 0.54 c	5.2	4.1
Threonine	4.94 ± 0.04 c	4.96 ± 0.07 c	4.50 ± 0.19 a	4.59 ± 0.07 ab	4.80 ± 0.41 bc	4.83 ± 0.03 bc	4.86 ± 0.08 c	4.83 ± 0.06 bc	4.93 ± 0.25 c	5.06 ± 0.25 c	3.1	2.5
Valine	5.93 ± 0.09 bc	6.04 ± 0.07 bc	5.56 ± 0.23 a	5.82 ± 0.10 ab	5.82 ± 0.53 ab	5.86 ± 0.05 abc	5.99 ± 0.10 bc	6.18 ± 0.05 c	5.79 ± 0.31 ab	6.00 ± 0.27 bc	4.3	4.0

RSP 1—recommended amino acid scoring pattern for children from 6 months to 3 years old; RSP 2—recommended amino acid scoring pattern for older children, adolescents, and adults; one-factor analysis of variance and Fisher post hoc test were applied. Data are shown as the mean ± SEM. Mean values within a row followed by different letters differ significantly (*p* ≤ 0.05).

## Data Availability

The data used to support the findings of this study can be made available from the corresponding author upon request.

## Supplementary Materials

The following supporting information can be downloaded at: https://www.mdpi.com/article/10.3390/foods11233762/s1, Table S1: The antioxidant potential of fermented moringa leaves is influenced by fungal strain and fermentation time; Figure S1: Base peak chromatogram of phenolic compounds analysed using HPLC-ESI-TOF-MS; Figure S2: Representative chromatogram of amino acid analysis of moringa leaves fermented for 3 days with *Neurospora intermedia*.
