# Supplementary material for: Phenolic Profile, Antioxidant Activity and Amino Acid Composition of Moringa Leaves Fermented with Edible Fungal Strains
Source: Foods. 2022 Nov 22;11(23):3762. doi: 10.3390/foods11233762 (PMC9736034; doi:10.3390/foods11233762)
Supplement: Supplementary file 1 [file foods-11-03762-s001.zip › foods-2005389-supplementary.pdf]

Table S1. The antioxidant potential of fermented moringa leaves as influenced by fungal strain and fermentation time.

	**FCRS (mg/g DM)**	**SA-ABTS$^{\cdot+}$ (µmol trolox/g DM)**	**SA-$^{\cdot}$OH (IC_{50})**
Factor: strain			
Rhizopus oryzae CBS 372.63	31.25±7.14 d	345.77±70.27 c	0.90±0.08 a
Rhizopus oligosporus ATCC 64063	27.63±8.24 c	336.37±127.26 b	1.09±0.15 b
Aspergillus oryzae CBS 673.92	26.14±7.19 b	337.09±128.64 b	1.20±0.09 c
Neurospora intermedia CBS 131.92	25.67±8.80 a	283.93±69.11 a	1.11±0.17 b
Factor: time (days)			
1	40.10±1.98 d	486.07±64.60 d	1.17±0.15 b
3	26.26±4.01 c	296.69±36.40 c	1.02±0.18 a
8	23.08±2.42 b	268.40±26.73 b	1.05±0.13 a
16	21.24±3.03 a	251.99±34.12 a	1.07±0.18 a

Two-factor analysis of variance and Fisher post-hoc test were applied. Data is shown as the mean ± SE. Mean values within a column followed by different letters differ significantly ($p \leq 0.05$) within a factor. FCRS - Folin-Ciocalteu reacting substances; SA-ABTS$^{\cdot+}$ - ABTS$^{\cdot+}$-scavenging activity; SA-$^{\cdot}$OH - $^{\cdot}$OH-scavenging activity; IC_{50}: Half maximal inhibitory concentration.

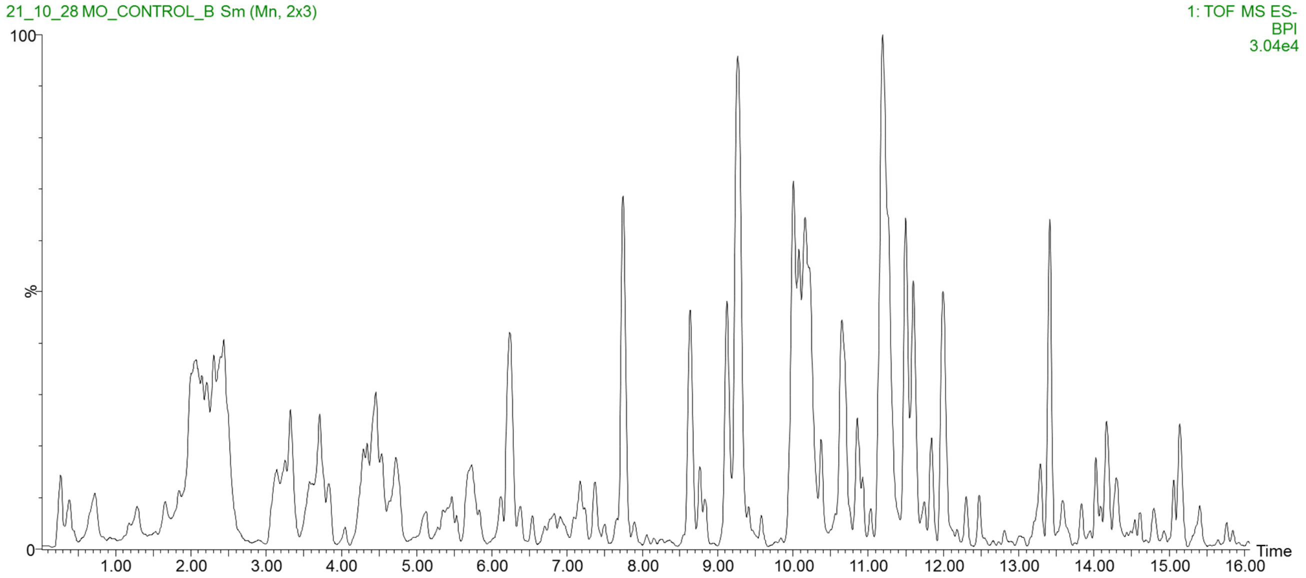

Figure S1. Base peak chromatogram of phenolic compounds analysed by HPLC-ESI-TOF-MS.

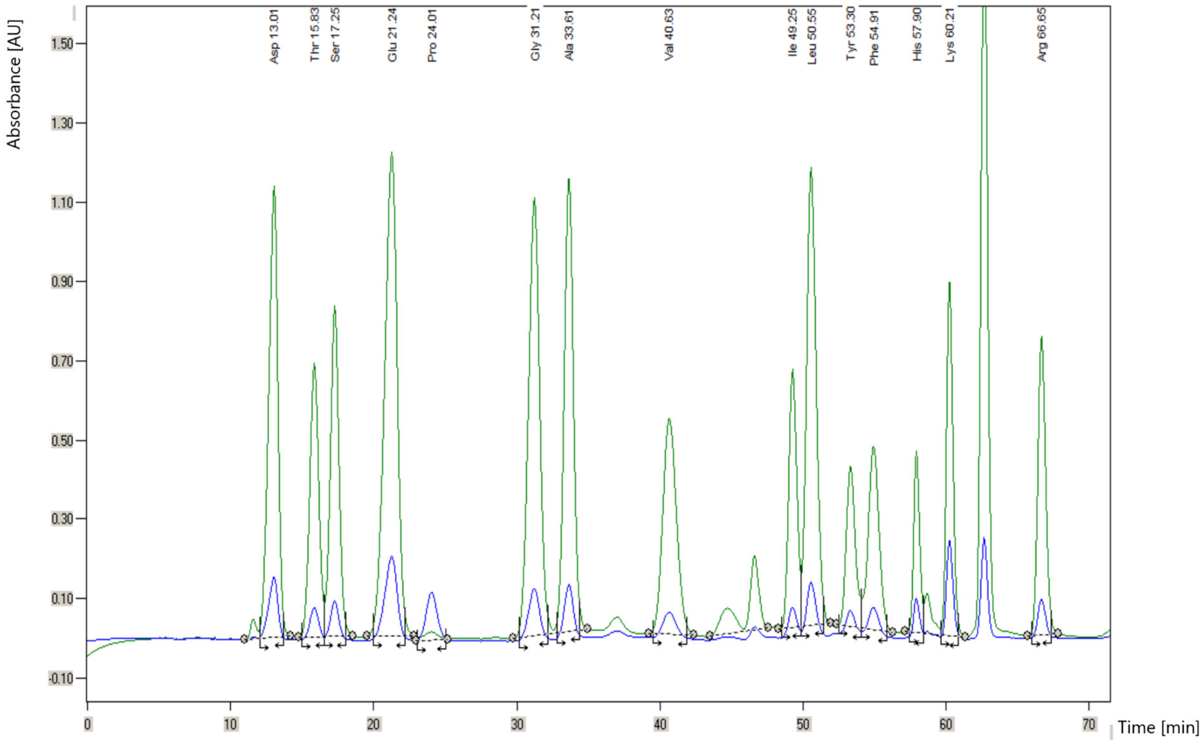

Figure S2. Representative chromatogram of amino acid analysis - moringa leaves fermented for 3 days with *Neurospora intermedia*. Green line - detection wavelength 570 nm, blue line - detection wavelength 440 nm.
